# Stability of the Associations between Early Life Risk Indicators and Adolescent Overweight over the Evolving Obesity Epidemic

**DOI:** 10.1371/journal.pone.0095314

**Published:** 2014-04-18

**Authors:** Lise Graversen, Thorkild I. A. Sørensen, Liselotte Petersen, Ulla Sovio, Marika Kaakinen, Annelli Sandbæk, Jaana Laitinen, Anja Taanila, Anneli Pouta, Marjo-Riitta Järvelin, Carsten Obel

**Affiliations:** 1 Section for General Medical Practice, Department of Public Health, Aarhus University, Aarhus, Denmark; 2 Institute of Preventive Medicine, Bispebjerg and Frederiksberg University Hospital, The Capital Region, Copenhagen, Denmark; 3 Novo Nordisk Foundation Center for Basic Metabolic Research, Faculty of Health and Medical Sciences, University of Copenhagen, Copenhagen, Denmark; 4 National Centre for Register-Based Research, Faculty of Social Sciences, Aarhus University, Aarhus, Denmark; 5 Department of Obstetrics and Gynaecology, University of Cambridge, Cambridge, United Kingdom; 6 Department of Epidemiology and Biostatistics, Imperial College, London, United Kingdom; 7 Institute of Health Sciences, University of Oulu, Oulu, Finland; 8 Biocenter Oulu, University of Oulu, Oulu, Finland; 9 Finnish Institute of Occupational Health, Oulu, Finland; 10 Primary Health Care Unit, University Hospital of Oulu, Oulu, Finland; 11 National Institute of Health and Welfare, Oulu, Finland; 12 Department of Obstetrics and Gynecology, University of Oulu and Oulu University Hospital, Oulu, Finland; National Taiwan University Hospital, Taiwan

## Abstract

**Background:**

Pre- and perinatal factors and preschool body size may help identify children developing overweight, but these factors might have changed during the development of the obesity epidemic.

**Objective:**

We aimed to assess the associations between early life risk indicators and overweight at the age of 9 and 15 years at different stages of the obesity epidemic.

**Methods:**

We used two population-based Northern Finland Birth Cohorts including 4111 children born in 1966 (NFBC1966) and 5414 children born in 1985–1986 (NFBC1986). In both cohorts, we used the same a priori defined prenatal factors, maternal body mass index (BMI), birth weight, infant weight (age 5 months and 1 year), and preschool BMI (age 2–5 years). We used internal references in early childhood to define percentiles of body size (<50, 50–75, 75–90 and >90) and generalized linear models to study the association with overweight, according to the International Obesity Taskforce (IOTF) definitions, at the ages of 9 and 15 years.

**Results:**

The prevalence of overweight at the age of 15 was 9% for children born in 1966 and 16% for children born in 1986. However, medians of infant weight and preschool BMI changed little between the cohorts, and we found similar associations between maternal BMI, infant weight, preschool BMI, and later overweight in the two cohorts. At 5 years, children above the 90^th^ percentile had approximately a 12 times higher risk of being overweight at the age of 15 years compared to children below the 50^th^ percentile in both cohorts.

**Conclusions:**

The associations between early body size and adolescent overweight showed remarkable stability, despite the increase in prevalence of overweight over the 20 years between the cohorts. Using consequently defined internal percentiles may be a valuable tool in clinical practice.

## Introduction

The prevalence of overweight especially among children in the developed countries has been increasing for decades [1], [2]. Childhood overweight and obesity have serious public health implications, as they are linked to adverse health outcomes in childhood, and they track into adulthood [3]–[7]. However, the prevention of weight gain and the management of established overweight pose major challenges. Interventions to treat overweight in children have shown small effects [8], [9] and only a few studies of interventions to prevent childhood overweight have been performed. However, recent reviews indicate that intervention in the preschool years involving the parents [9] may have larger effect than intervention in later childhood [10]. The identification of preschool children at risk of developing overweight followed by well-designed preventive interventions seems therefore highly warranted.

Pre- and perinatal risk indicators and preschool measures of body size have been studied in relation to later overweight [11]–[15], and the association with later overweight is confirmed for a number of risk indicators, such as high maternal body mass index (BMI (kg/m^2^)) [16]–[19], parity [20], parental level of education [21], birth weight [22]–[25], different measures of infancy/preschool weight, BMI or weight gain [13], [26]–[30], and early BMI rebound (the age when BMI reaches its nadir) [31], [32].

In many countries, data on these risk indicators is available, and height and weight are measured when preschool children and their parents attend routine health examinations by pediatricians or general practitioners.

A more targeted use of these early life risk indicators in the prevention of obesity requires a study to clarify, whether the rising prevalence of overweight is linked to 1) a rise in the levels of early life risk indicators, or 2) a stronger association between upper levels of early life risk indicators and later overweight, or 3) whether the rise in overweight prevalence in adolescence/adulthood is a general phenomenon affecting the population throughout the broad range of early life risk indicators. The Northern Finland Birth Cohorts offer a unique opportunity of studying cohorts from different stages of the obesity epidemic including this broad range of risk indicators.

The aim of the present study was to test the associations between pre- and perinatal risk indicators, simple postnatal measures of body size up till the age of 5 years (infant weight (5 months and 1 year) and preschool BMI (2–5 years)) and later overweight. The study compares the associations in two large population-based cohorts of children born 20 years apart, i.e. at different stages of the obesity epidemic. We compared the cohorts at an as advanced age as possible (the age of 15 years), but as growth spurts in adolescence alter growth development and could confound the findings, we also compared the cohorts before adolescence (at the age of 9 years).

## Material and Methods

### NFBC1966

The Northern Finland Birth Cohort 1966 (NFBC1966) consists of 96.3% of all children who were due to be born in the provinces of Oulu and Lapland in Northern Finland in 1966, and 11 744 live-born singletons entered the study [33], [34]. Data collection was started in pregnancy via a structured, self-completed questionnaire concerning health and the family's social situation. Data on pregnancy and birth were collected prospectively. Data on postnatal growth up till adolescence was obtained from scanning the original health clinic records (N = 4310). Children born before the 36^th^ gestational week were excluded from the present study. Full antenatal data and postnatal growth data were available for 4111 singletons in the NFBC1966 cohort. Sufficient data to estimate growth curves was available for 2120 children. BMI was available for 1399 children at the age of 9 years, and for 1911 children at the age of 15 years (Table 1).

**Table 1:** Medians (5% and 95% percentiles) and percentages of outcomes and risk indicators in the NFBC1966 and NFBC1986.

	NFBC1966 (N = 4111)		NFBC1986 (N = 5414)	
	N	Median (5–95% percentile)	N	Median (5–95% percentile)
**Outcome 9 years**				
BMI (kg/m^2^)**	1399	16.2(14.0–21.0)	4064	16.7(14.2–22.3)
Overweight/obese according to IOTF (%)	1399	9%	4064	17%
**Outcome 15 years**				
BMI (kg/m^2^)**	1911	19.5(16.5–25.2)	3709	20.2(16.9–27.3)
Overweight/obese according to IOTF (%)	1911	9%	3709	16%
**Risk indicators**				
Maternal BMI (kg/m^2^)**	1985	22.3(18.8–28.6)	4480	21.6(18.3–28.7)
Maternal smoking 1–10 cig/day	2079	12%	4489	9%
Maternal smoking >10 cig/day	2079	2%	4489	9%
Maternal cohabiting (%)	2118	97%	4572	96%
Maternal education longer than primary school	2090	38%	4049	76%
Maternal age (years)**	2117	26.1(19.1–38.6)	4581	27.9(20.5–38.3)
Offspring first born (%)	2117	42%	4565	34%
Offspring male (%)	2120	49%	4581	49%
Birth weight (g)**	2120	3515(2780–4300)	4581	3600(2840–4420)
Weight at 5 months (kg)**	2120	7.4(6.2–8.9)	4581	7.3(6.1–8.8)
Weight at 1 year (kg)**	2120	10.2(8.6–12.2)	4581	9.9(8.3–11.9)
BMI at 2 years (kg/m^2^)**	2120	16.7(14.7–18.9)	4581	16.5(14.7–18.7)
BMI at 3 years (kg/m^2^)	2120	16.1(14.2–18.3)	4581	16.0(14.3–18.2)
BMI at 4 years (kg/m^2^)**	2120	15.7(13.9–17.9)	4581	15.7(14.1–18.0)
BMI at 5 years (kg/m^2^)**	2120	15.4(13.5–18.0)	4581	15.5(13.8–18.2)

**Statistically significant difference between cohorts. BMI- Body mass index. IOTF – overweight/obese according to the International Obesity Task Force. NFBC1966 and NFBC1986 – Northern Finnish Birth Cohorts born in 1966 and 1986.

### NFBC1986

The Northern Finland Birth Cohort 1986 (NFBC1986) consists of 99% of all children who were due to be born in the provinces of Oulu and Lapland in Northern Finland between 1 July 1985 and 30 June 1986, and 9203 live-born singletons entered the study. Data collection and inclusion criteria were similar to the NFBC1966. Data on postnatal growth were available for 5674 children. Full antenatal data and data on postnatal growth were available for 5414 singletons in the NFBC1986 cohort. Sufficient data to estimate growth curves was available for 4581 children. BMI was available for 4064 at the age of 9 years, and for 3709 at the age of 15 years (Table 1).

Both study populations were homogenous in terms of ethnicity.

### Ethics statement

Signed, informed consent and written permission to use their data for scientific research was obtained from the study participants at the age of 31 in the NFBC1966. In the NFBC1986, the adolescents and their parents gave informed consent and written permission to use their data for scientific research. The University of Oulu Ethics Committee approved the study.

### Measures of growth

Birth weight was obtained from medical records. Birth weight was divided into percentile groups according to week of gestation from the 36^th^ to the 43^rd^ week. From birth till the age of 5.5 years, individual growth curves were fitted (File S1), and weight and height at specific time points were extracted from these fitted growth curves. From birth until 1 year of age, we used weight, and from the age of 2 to 5 years, we used BMI, as these measures of body size are generally used in clinical practice. We subdivided children at these specific time points into 4 groups according to weight or BMI percentiles (<50, 50–75, 75–90 and >90) in the study population. Participants were categorized at the age of 9 and 15 years as normal weight or overweight (including obese) using the BMI cut-offs recommended by the Childhood Obesity Working Group of the International Obesity Taskforce (IOTF) [35].

### Statistical analyses

Differences in the examined weight and BMI medians and distributions between the cohorts were tested using Wilcoxon rank-sum test. A 5% significance level was used.

The relative risk of overweight at the age of 15 years (defined as the latest measurement between the age of 14 and 16 years) by different risk indicators were analyzed using a generalized linear regression model with log link. In adolescence, sexual maturation causes a growth spurt. We tested the associations with overweight just before adolescence, i.e. at the age of 9 years (latest measurement between the age of 8 and 10 years) to account for differences in pubertal development between the cohorts. Moreover, we tested for interactions with gender; in the NFBC1986, BMI at the age of 2 years was more strongly associated with overweight at the age of 9 years in females than in males. At the age of 15 years, this was the case for BMI from 2 to 4 years. As this was not the case in general, and because differences in associations between the genders decreased, when overweight was examined at the age of 16 years, the associations shown are not stratified, but adjusted for gender.

To explore whether slightly preterm babies or babies with fetal growth retardation had an impact on the results, we also performed the analysis excluding infants with birth weight <2500 g, infants small for gestational age (the lowest 5% for every gestational week) and infants born in the 36^th^ week of gestation.

We calculated predictive values of the strongest risk indicators. The predictive values are presented as sensitivity, specificity, positive predictive value (PPV) and negative predictive value (NPV).

Stata version 10 SE was used for all statistical analyses. The results are reported as medians (with the 5–95% percentiles), percentages and relative risks (with their 95% confidence intervals (CI)).

## Results

The descriptive data of the two cohorts are displayed in Table 1. The prevalence of overweight, measured at the age of 9 and 15 years, was 9% for both ages in the NFBC1966, and 17% at the age of 9, and 16% at the age of 15 in the NFBC1986. Medians of infant weight (5 months and 1 year) and preschool BMI (2–5 years) in the two cohorts were overall similar, but small differences were detected.

Surprisingly, the median maternal BMI fell slightly from the NFBC1966 to the NFBC1986. Further analysis revealed an equal median weight, but an increased median height in the NFBC1986 compared with the NFBC1966.

In Tables 2 and 3, we present infant weight and preschool BMI and the corresponding risk of overweight at the age of 9 and 15 years. We tested for differences between the cohorts using a 5% significance level. Very few differences were found, and we therefore also calculated common estimates for the two cohorts by pooling all the data. Excluding slightly preterm babies and babies with fetal growth retardation did not change the results.

**Table 2:** Relative risk of overweight at the age of 9 years according to the IOTF associated with early weight and BMI measures in the NFBCC1966 and the NFBC1986.

		NFBC1966 (N = 1399)			NFBC1986 (N = 4064)		Total (N = 5463)		
	N	n(%)	RR(95%CI)	N	n(%)	RR(95%CI)	N	n(%)	RR(95%CI)
Weight at 5 months (kg)									
<50 percentile	685	55(8)	1	2038	227(11)	1	2723	282(10)	1
≥50–<75	355	29(8)	1.2(0.7–1.8)	1013	192(19)	1.9(1.6–2.2)	1368	221(16)	1.7(1.5–2.0)
≥75–<90	215	20(9)	1.4(0.9–2.4)	610	129(21)	2.2(1.8–2.6)	825	149(18)	2.0(1.7–2.4)
≥90	144	23(16)	2.6(1.6–4.1)	403	125(31)	3.2(2.6–4.0)	547	148(27)	3.1(2.6–3.7)
Weight at 1 year (kg)									
<50 percentile	699	52(7)	1	2031	210(10)	1	2730	262(10)	1
≥50–<75	345	27(8)	1.2(0.7–1.8)	1016	188(19)	1.9(1.6–2.3)	1361	215(16)	1.8(1.5–2.1)
≥75–<90	210	21(10)	1.6(1.0–2.6)	608	143(24)	2.5(2.1–3.0)	818	164(20)	2.3(1.9–2.8)
≥90	145	27(19)	3.0(2.0–4.6)	409	132(32)	3.5(2.9–4.3)	554	159(29)	3.4(2.8–4.0)
BMI at 2 years (kg/m^2^)									
<50 percentile	701	39(6)	1	2036	196(10)	1*	2737	235(9)	1
≥50–<75	353	31(9)	1.6(1.0–2.6)	1007	161(16)	1.7(1.4–2.0)	1360	192(14)	1.7(1.4–2.0)
≥75–<90	202	23(11)	2.1(1.3–3.5)	617	147(24)	2.5(2.1–3.0)	819	170(21)	2.5(2.1–3.0)
≥90	143	34(24)	4.5(3.0–6.9)	404	169(42)	4.4(3.7–5.3)	547	203(37)	4.4(3.8–5.2)
BMI at 3 years (kg/m^2^)									
<50 percentile	710	25(4)	1	2028	141(7)	1	2738	166(6)	1
≥50–<75	345	32(9)	2.7(1.7–4.5)	1024	171(17)	2.4(2.0–3.0)	1369	203(15)	2.5(2.0–3.0)
≥75–<90	206	28(14)	4.0(2.4–6.7)	615	152(25)	3.6(2.9–4.5)	821	180(22)	3.7(3.0–4.5)
≥90	138	42(30)	8.9(5.6–14.0)	397	209(53)	7.6(6.3–9.2)	535	251(47)	7.8(6.6–9.3)
BMI at 4 years (kg/m^2^)									
<50 percentile	706	18(3)	1	2030	83(4)	1	2736	101(4)	1
≥50–<75	341	19(6)	2.3(1.2–4.3)	1024	153(15)	3.7(2.8–4.7)	1365	172(13)	3.4(2.7–4.4)
≥75–<90	217	32(15)	5.9(3.4–10.2)	607	178(29)	7.2(5.7–9.2)	824	210(25)	7.0(5.6–8.7)
≥90	135	58(43)	17.1(10.4–27.9)	403	259(64)	15.7(12.6–19.7)	538	317(59)	16.0(13.1–19.6)
BMI at 5 years (kg/m^2^)									
<50 percentile	697	14(2)	1	2038	62(3)	1	2735	76(3)	1*
≥50–<75	348	24(7)	3.5(1.9–6.7)	1010	135(13)	4.4(3.2–5.9)	1358	159(12)	4.2(3.2–5.5)
≥75–<90	205	25(12)	6.2(3.3–11.8)	603	195(32)	10.6(8.1–14.0)	808	220(27)	9.9(7.7–12.6)
≥90	149	64(43)	21.0(12.1–36.4)	413	281(68)	22.3(17.3–28.8)	562	345(61)	22.0(17.5–27.8)

*Statistically significant difference between genders. No statistically significant differences between the cohorts found. BMI- Body mass index. IOTF – overweight/obese according to the International Obesity Task Force. NFBC1966 and NFBC1986 – Northern Finnish Birth Cohorts born in 1966 and 1986.

**Table 3:** Relative risk of overweight at the age of 15 years according to the IOTF associated with early weight and BMI measures in the NFBC1966 and the NFBC1986.

		NFBC66 (N = 1911)			NFBC86 (N = 3709)		Total(N = 5620)		
	N	n(%)	RR(95%CI)	N	n(%)	RR(95%CI)	N	n(%)	RR(95%CI)
Weight at 5 months (kg)									
<50 percentile	949	65(7)	1	1878	198(11)	1	2827	263(9)	1
≥50–<75	482	52(11)	1.7(1.2–2.4)	931	175(19)	1.7(1.4–2.1)	1413	227(16)	1.7(1.5–2.0)
≥75–<90	290	24(8)	1.4(0.9–2.2)	547	109(20)	1.8(1.5–2.3)	837	133(16)	1.7(1.4–2.1)
≥90	190	26(14)	2.3(1.5–3.6)	353	95(27)	2.4(1.9–3.0)	543	121(22)	2.4(1.9–2.9)
Weight at 1 year (kg)									
<50 percentile	955	62(6)	1	1868	196(10)	1	2823	258(9)	1
≥50–<75	483	46(10)	1.6(1.1–2.4)	931	158(17)	1.6(1.3–1.9)	1414	204(14)	1.6(1.3–1.9)
≥75–<90	284	26(9)	1.6(1.0–2.4)	551	121(22)	2.0(1.6–2.5)	835	147(18)	1.9(1.6–2.3)
≥90	189	33(17)	3.0(2.0–4.5)	359	102(28)	2.6(2.1–3.2)	548	135(25)	2.7(2.2–3.2)
BMI at 2 years (kg/m^2^)									
<50 percentile	955	48(5)	1	1872	174(9)	1*	2827	222(8)	1
≥50–<75	477	37(8)	1.6(1.0–2.4)	918	141(15)	1.6(1.3–2.0)	1395	178(13)	1.6(1.3–1.9)
≥75–<90	284	46(16)	3.3(2.2–4.8)	563	135(24)	2.5(2.0–3.1)	847	181(21)	2.7(2.2–3.2)
≥90	195	36(18)	3.7(2.5–5.6)	356	127(36)	3.7(3.0–4.5)	551	163(30)	3.7(3.1–4.4)
BMI at 3 years (kg/m^2^)									
<50 percentile	954	35(4)	1	1870	131(7)	1*	2824	166(6)	1*
≥50–<75	479	42(9)	2.5(1.6–3.8)	919	150(16)	2.3(1.9–2.9)	1398	192(14)	2.3(1.9–2.8)
≥75–<90	279	39(14)	3.9(2.5–6.0)	565	144(25)	3.5(2.9–4.4)	844	183(22)	3.6(3.0–4.4)
≥90	199	51(26)	7.0(4.7–10.4)	355	152(43)	6.0(4.9–7.3)	554	203(37)	6.2(5.1–7.4)
BMI at 4 years (kg/m^2^)									
<50 percentile	955	23(2)	1	1868	97(5)	1*	2823	120(4)	1
≥50–<75	475	37(8)	3.3(2.0–5.5)	930	144(15)	3.0(2.3–3.8)	1405	181(13)	3.0(2.4–3.8)
≥75–<90	287	40(14)	5.8(3.6–9.6)	549	149(27)	5.1(4.0–6.5)	836	189(23)	5.3(4.2–6.5)
≥90	194	67(35)	14.3(9.2–22.4)	362	187(52)	9.7(7.9–12.2)	556	254(46)	10.7(8.8–13.0)
BMI at 5 years (kg/m^2^)									
<50 percentile	952	24(3)	1	1876	89(5)	1	2828	113(4)	1
≥50–<75	475	31(7)	2.6(1.6–4.4)	920	129(14)	2.9(2.3–3.8)	1395	160(11)	2.8(2.3–3.6)
≥75–<90	290	45(16)	6.2(3.9–10.1)	549	165(30)	6.2(4.9–7.9)	839	210(25)	6.2(5.0–7.7)
≥90	194	67(35)	13.5(8.7–21.0)	364	194(53)	11.1(8.9–13.9)	558	261(47)	11.7(9.6–14.3)

*Statistically significant difference between genders. No statistically significant differences between the cohorts found. BMI- Body mass index. IOTF – overweight/obese according to the International Obesity Task Force. NFBC1966 and NFBC1986 – Northern Finnish Birth Cohorts born in 1966 and 1986.

Infant weight and preschool BMI were strongly associated with overweight at the age of 9 and 15 years in both cohorts. As expected, the strength of the associations weakened with increasing time between exposure and outcome.

In Tables 4 and 5, we present pre- and perinatal risk indicators and the corresponding risk of overweight at the age of 9 and 15 years. High and low maternal age only seemed to be associated with offspring overweight in the NFBC1966. Female gender was associated with a higher risk of overweight than male gender in the NFBC1966 at the age of 9 years and 15 years. However, in the NFBC1986, there was no difference at the age of 9 years, but at the age of 15 years, we found that males were more likely to be overweight than females. All other factors were similarly associated in the two cohorts. Maternal BMI was associated with overweight at both ages in both cohorts with similar strength. Birth weight, maternal smoking, and single motherhood all seemed to be associated with overweight, but the associations were not consistently statistically significant.

**Table 4:** Relative risk of overweight at the age of 9 years according to the IOTF associated with pre- and perinatal risk indicators in the NFBC1966 and the NFBC1986.

	NFBC1966 (N = 1399)			NFBC1986 (N = 4064)			Total(N = 5463)		
	N	n(%)	RR(95%CI)	N	n(%)	RR(95%CI)	N	n(%)	RR(95%CI)
Maternal BMI (kg/m^2^)									
<25	1042	83(8)	1	3334	474(14)	1	4376	557(13)	1
≥25–<30	225	30(13)	1.6(1.1–2.4)	512	132(26)	1.8(1.5–2.1)	737	162(22)	1.7(1.5–2.0)
≥30	39	9(23)	2.9(1.6–5.3)	133	51(38)	2.7(2.1–3.4)	172	60(35)	2.7(2.2–3.4)
Maternal smoking									
no	1187	104(9)	1	3275	489(15)	1	4462	593(13)	1
1–10 cigarettes/day	159	16(10)	1.2(0.7–1.9)	338	77(23)	1.5(1.2–1.9)	497	93(19)	1.4(1.2–1.7)
>10 cigarettes/day	31	5(16)	2.0(0.9–4.4)	371	97(26)	1.8(1.5–2.1)	402	102(25)	1.9(1.6–2.3)
Maternal cohabiting									
Cohabiting	1358	120(9)	1	3893	639(16)	1	5251	759(14)	1
Single	39	6(15)	1.6(0.8–3.5)	165	34(21)	1.3(0.9–1.7)	204	40(19)	1.3(1.0–1.8)
Maternal age									
<25	597	58(10)	1.3(0.9–1.8)	1135	183(16)	1.0(0.8–1.1)	1732	241(14)	0.9(0.8–1.1)
25–35	611	46(8)	1	2412	400(17)	1	3023	446(15)	1
>35	190	23(12)	1.6(1.0–2.6)	517	90(17)	1.1(0.9–1.3)	707	113(16)	1.1(0.9–1.3)
Long maternal education									
no	858	76(9)	1	859	160(19)	1	1717	236(14)	1
yes	525	50(10)	1.1(0.8–1.5)	2793	451(17)	0.9(0.8–1.0)	3259	501(15)	1.1(1.0–1.3)
Offspring gender									
Male	670	46(7)	1	1999	325(16)	1	2669	371(14)	1**
Female	729	81(11)	1.6(1.1–2.3)	2065	348(17)	1.0(0.9–1.2)	2794	429(15)	1.1(1.0–1.3)
Birth weight (kg)									
<50 percentile	708	65(9)	1	2046	289(14)	1	2754	354(13)	1
≥50–<75	362	22(6)	0.7(0.4–1.1)	1002	154(15)	1.1(0.9–1.3)	1364	176(13)	1.0(0.9–1.2)
≥75–<90	187	24(13)	1.5(0.9–2.3)	620	124(20)	1.4(1.2–1.7)	807	148(18)	1.5(1.2–1.7)
≥90	142	16(11)	1.3(0.8–2.2)	396	106(27)	1.9(1.6–2.4)	538	122(23)	1.8(1.5–2.2)
Offspring first born									
yes	576	56(10)	1	1341	246(18)	1	1917	302(16)	1
no	820	71(9)	0.9(0.6–1.2)	2712	425(16)	0.9(0.9–1.0)	3532	496(14)	0.9(0.8–1.0)

No statistically significant differences between genders found.

**Statistically significant difference between cohorts. BMI- Body mass index. IOTF – overweight/obese according to the International Obesity Task Force. NFBC1966 and NFBC1986 – Northern Finnish Birth Cohorts born in 1966 and 1986.

**Table 5:** Relative risk of overweight at the age of 15 years according to the IOTF associated with pre-and perinatal risk indicators in the NFBC1966 and the NFBC1986.

	NFBC1966 (N = 1911)			NFBC1986 (N = 3709)			Total(N = 5620)		
	N	n(%)	RR(95%CI)	N	n(%)	RR(95%CI)	N	n(%)	RR(95%CI)
Maternal BMI (kg/m^2^)									
<25	1456	104(7)	1	3054	392(13)	1	4510	496(11)	1
≥25–<30	297	45(15)	2.1(1.5–2.9)	454	115(25)	2.0(1.6–2.3)	751	160(21)	1.9(1.7–2.3)
≥30	44	12(27)	3.9(2.3–6.4)	120	52(43)	3.4(2.7–4.2)	164	64(39)	3.5(2.9–4.4)
Maternal smoking									
no	1629	138(8)	1	2205	439(15)	1	4634	577(12)	1
1–10 cigarettes/day	210	24(11)	1.3(0.9–2.0)	303	61(20)	1.4(1.1–1.8)	513	85(17)	1.3(1.1–1.6)
>10 cigarettes/day	35	2(6)	0.7(0.2–2.7)	328	66(20)	1.4(1.1–1.7)	363	68(19)	1.5(1.2–1.9)
Maternal cohabiting									
Cohabiting	1857	161(9)	1	3548	551(16)	1	5405	712(13)	1
Single	52	6(12)	1.3(0.6–2.8)	156	26(17)	1.1(0.8–1.6)	208	32(15)	1.2(0.9–1.7)
Maternal age									
<25	832	74(9)	1.3(0.9–1.7)	1046	162(15)	1.0(0.8–1.2)	1878	236(13)	1.0(0.9–1.2)
25–35	848	60(7)	1	2190	340(16)	1	3038	400(13)	1**
>35	228	32(14)	2.0(1.3–3.0)	473	75(16)	1.0(0.8–1.3)	701	107(15)	1.2(1.0–1.5)
Long maternal education									
no	1158	112(10)	1	788	136(17)	1	1946	248(13)	1
yes	725	53(7)	0.8(0.6–1.0)	2504	390(16)	0.9(0.8–1.1)	3229	443(14)	1.1(0.9–1.2)
Offspring gender									
Male	920	70(8)	1	1815	332(18)	1	2735	4202(15)	1**
Female	991	97(10)	1.3(1.0–1.7)	1894	245(13)	0.7(0.6–0.8)	2885	342(12)	0.8(0.7–0.9)
Birth weight (g)									
<50 percentile	973	85(9)	1	1872	236(13)	1	2845	321(11)	1
≥50–<75	491	38(8)	0.9(0.6–1.3)	932	153(16)	1.3(1.0–1.5)	1423	191(13)	1.2(1.0–1.4)
≥75–<90	266	25(9)	1.1(0.7–1.7)	556	104(19)	1.4(1.1–1.7)	822	129(16)	1.4(1.1–1.6)
≥90	181	19(11)	1.3(0.8–2.0)	349	84(24)	1.8(1.4–2.2)	530	103(19)	1.7(1.4–2.0)
Offspring first born									
yes	789	71(9)	1	1229	198(16)	1	2018	269(13)	1
no	1119	96(9)	1.0(0.7–1.3)	2468	377(15)	1.0(0.8–1.1)	3587	473(13)	1.0(0.9–1.1)

No statistically significant differences between genders found.

**Statistically significant difference between cohorts. BMI- Body mass index. IOTF – overweight/obese according to the International Obesity Task Force. NFBC1966 and NFBC1986 – Northern Finnish Birth Cohorts born in 1966 and 1986.

Positive predictive values for all risk indicators can be seen in the tables 2–5 (n(%)) and full predictive values of being among the top 10 per cent in BMI at the age of 5 years or being exposed to maternal pre-pregnancy overweight (incl. obesity) are shown in table 6. The PPV of being overweight at the age of 15 years linked to being among the top 10% in preschool growth increased with age (table 2 and 3). Furthermore, the PPV increases from the NFBC1966 to the NFBC1986 due to increasing overweight prevalence. The PPV reached 53% at the age of 5 years in the NFBC1986 meaning that half of all children in the top 10% of BMI at the age of 5 years are overweight at the age of 15 years.

**Table 6:** Predictive values of 5 year BMI in the top 10% and maternal overweight (incl. obesity).

Predicting overweight at 15 years	Sensitivity	Specificity	PPV	NPV
5 year BMI in the top 10%, NFBC1966	40.1	92.7	34.5	94.2
5 year BMI in the top 10%, NFBC1986	33.6	94.6	53.3	88.6
Maternal overweight (incl. obesity),NFBC1966	35.4	82.6	16.7	92.9
Maternal overweight (incl. obesity), NFBC1986	29.9	86.7	29.1	87.2

BMI- Body mass index. PPV- positive predictive value. NPV - negative predictive value. NFBC1966 and NFBC1986 – Northern Finnish Birth Cohorts born in 1966 and 1986.

## Discussion

### Main findings

In this study, we examined how predefined pre- and perinatal risk indicators, infant weight, and preschool BMI were associated with overweight at the age of 9 and 15 years. We studied this in two large longitudinal birth cohorts born in 1966 and 1986. We found that maternal BMI, infant weight, and preschool BMI were strongly associated with overweight just before and during adolescence in both cohorts. We found no substantial differences between the two cohorts in terms of changes in median infant weight or preschool BMI, and the relative risk associated with the percentile division of these were very similar in the two cohorts. A third of all children in the top 10% of 5 year BMI were overweight at the age of 15 years in the NFBC1966, and this was the case for more than half of children in the top 10% of 5 year BMI in the NFBC1986.

### Comparison with other studies

The associations between overweight in later life and early life risk indicators, such as parity [36], education level [36], maternal smoking during pregnancy [36]–[38], maternal pre-pregnancy BMI [36], [38], [39], and birth weight [37], [38] in the Northern Finnish Birth Cohorts have previously been reported with more statistical power owing to larger sample sizes, but a comparison of the association with adolescence overweight in the two cohorts has not previously been reported. Few studies have the opportunity to examine the development of risk indicators over time. Rugholm et al [24] found that mean birth weight and the link between birth weight and school age overweight were stable over a 20-year period. We found a small increase in median birth weight from 1966 to 1986, but also failed to prove differences in the associations between birth weight and school age overweight between the cohorts. We confirmed the strong associations between weight and BMI measures early in life and school age BMI found in a number of studies [11]–[15], [26]–[30].

### Growth spurt in adolescence

We examined early life risk indicators in relation to overweight at the age of 9 years, as the growth spurt in adolescence could alter weight class at the age of 15 years. Age at growth spurt has decreased over time, and since heavier children are known to enter growth spurt earlier than other children [40], [41], this could influence our results. Hence, more children in the NFBC1986 than in the NFBC1966 were expected to have entered height or both height and weight spurt at the age of 15 years, because the proportion of heavy children was larger in 1986 than in 1966; these children could appear with a lower BMI due to this growth spurt. Girls are known to enter growth spurt earlier than boys [41] and in this study, we found girls to be more often normal weight than boys at the age of 15 years in the NFBC1986 (Table 5). Furthermore, the prevalence of overweight decreases slightly in the NFBC1986 from the age of 9 years to the age of 15 years. These findings could indicate that a lower BMI at the age of 15 years due to growth spurt could hide overweight children, especially girls, because they have entered growth spurt more frequently than their normal weight peers.

### BMI rebound

BMI rebound has been shown to be a strong indicator of adult overweight [32], but the clinical relevance of BMI rebound has been subject to doubt. Early BMI rebound may be an indicator of later obesity alone, because it identifies children, whose BMI percentile is high and/or crossing upwards [42]. Furthermore, weight and height measurements at the age of 8–9 years are needed to estimate BMI rebound.

We explored the possibility of estimating BMI rebound at the age of 5 years, as children with early rebound and hence at higher risk of overweight, will have had their BMI rebound before this age. Two strategies were tested: First, we defined BMI rebound as the lowest measured BMI between the age of 2 and 6 followed by higher measurements. Second, we defined it as the nadir on fitted BMI curves up till the age of 5.5, if a nadir existed. Both strategies failed to demonstrate stronger associations with future overweight than just BMI at the age of 5 years, even though children with no estimated BMI rebound were included as a separate group.

### Reference material and percentile division of risk indicators

Body size measures are most often defined according to one of various external reference materials [43], [44]. External references, if clearly defined, are an important epidemiological tool that may be used to follow the obesity epidemic. However, BMI distributions differ between countries and over time, and this has given rise to many different reference materials that are not necessarily the most adequate or clinically relevant reference materials for a given population [45]–[47].

In view of this, internal reference poses a valid alternative. The technical development of electronic patient files and central registration can today provide up-to-date reference material for a given population over a short period of time. Reference materials can be created by automated collection of growth measurements on all children attending e.g. preventive health examinations through childhood in a whole population. Of particular interest in a clinical setting is to identify children with the highest risk of obesity within a population. Thus, the internal reference material may be more appropriate for risk communication in a clinical setting than the external references many clinicians use today. This is the reason for our a priori decision of consequent use of internal percentiles in this study.

### Stability of risk indicators

The obesity epidemic, manifested in the rise in prevalence at the age of 9 and 15 years seen in the NFBC1986, does not seem to have much affected the median weight at 5 months and 1 year, and BMI from 2 to 5 years. However, the distribution of BMI is right skewing, and a predisposition to later overweight may have been established in some children with only small, or without changes in early life weight and BMI measures. As overweight is a slowly developing condition, we cannot reject that small changes seen in early childhood could reflect crucial changes in a child's growth trajectory. Alternatively, causes of the rising prevalence are to be found among factors affecting later childhood.

No major differences in the risk associated with percentile division of risk indicators were found between the two cohorts. This implies that the rise in overweight prevalence later in life is a general phenomenon that affects the entire population, throughout the broad range of risk indicators. Thus, the rise in prevalence of overweight cannot be attributed to a rise in levels of risk indicators or a greater effect of the upper levels of the risk indicators.

### Strengths and limitations

The strength of this study was the prospective data collection conducted in two large general population-based cohorts with extensive information about clinically relevant risk indicators. The children were followed prospectively from pregnancy until adolescence. Numerous height and weight measurements made it possible to fit growth curves and to estimate the size of the child at any point of time in early childhood. Moreover, the two cohorts, with individuals born 20 years apart, offered the unique possibility for studying risk indicators over time. One limitation was that some children had an insufficient number of measurements for growth modelling, restricting the study population size especially in the NFBC1966. The difference between the cohorts in the proportion with sufficient measurements is due to changes over the years in the timing of routine measurements. We know from other analyses of the representativeness of attendees that individuals with only basic education and individuals with unemployment history are slightly underrepresented among attendees [48], and that individuals with sufficient measurements to fit growth curves in the NFBC1966 have slightly lower adult BMI than individuals with insufficient numbers of measurements [49]. If this has impact on the results, we will, most likely, have underestimated the associations as the population studied is thought to be somewhat healthier than the total population. A more similar proportion with growth curves would most likely result in more similar associations between the cohorts rather than larger differences. Moreover, the lack of data on diet and physical activity collected at the same age in childhood could be seen as a limitation. Diet and physical activity patterns are likely to have changed over this 20-year period, but the evidence of these factors' association with overweight development in observational studies is inconsistent [50] and the probability that data on these factors would have changed the conclusion is minor. Finally, separate analysis of preterm infants was not possible due to the limited number of preterm infants.

## Conclusions

Infant weight and preschool BMI were strongly associated with overweight in later childhood and adolescence in both NFBC1966 and NFBC1986. Despite the substantial increase in the prevalence of overweight, the relative risk of overweight linked to the percentile division of early weight and BMI measures appeared to be stable in two cohorts born 20 years apart and, hence, at very different stages of the obesity epidemic. Infant weight and preschool BMI have great potential for clinical use in the prediction of a child's risk of developing overweight, as the relative risk linked to them remains stable over time. A child's risk of future overweight can be determined on the basis of its weight and BMI measures from early infancy, and throughout early childhood with even greater certainty. We may assume that this will also apply to future children and thus be beneficial as a component in early life prevention of overweight.

### Perspective

Providing physicians with knowledge about a specific child's risk of later overweight at routine health examinations could provide a unique opportunity for overweight prevention. However, in spite of good evidence of the tracking of overweight from adolescence to adulthood, some overweight adolescents become normal weight adults [51]. In order to make sure that we target individuals at risk of clinically relevant outcomes, further analyses of the associations between these preschool weight and BMI measures and adult outcomes linked to morbidity and mortality would be highly interesting.

## Supporting Information

File S1 Description of the growth modelling.
(DOCX)
